# Effect of empowerment: how and when do high-involvement work practices influence elder employees’ innovative performance?

**DOI:** 10.3389/fpsyg.2024.1336120

**Published:** 2024-02-05

**Authors:** Daokui Jiang, Yiting Zhang, Honghong Zhu, Xiaoyu Wang

**Affiliations:** ^1^Business School, Shandong Normal University, Jinan, China; ^2^Institute of International Studies, Fudan University, Shanghai, China

**Keywords:** high-involvement work practices, innovation performance, exploratory innovation, exploitative innovation, transformational leadership

## Abstract

**Introduction:**

In today’s fast-paced business environment, innovation from elder employees is increasingly vital to organizations. High-involvement work practices that emphasize engagement and empowerment have a significant impact on the innovation performance of these employees, harnessing their wealth of experience and fostering organizational growth. However, most of the current research on innovation performance focuses on the single factor of the individual or the organization, and most of them focus on the linear relationship; research on the factor of human resource practices, in particular high-involvement work practices, is inadequate.

**Methods:**

Based on social exchange theory, this paper uses structural equation modeling (SEM) to examine the impact of high-involvement work practices on elder workers’ innovation performance using 278 valid samples from three time points, and the non-linear effects of exploratory and exploitative innovation on elder workers’ innovation performance.

**Results:**

(1) There is no significant relationship between high-involvement work practices and elder employees’ innovation performance. (2) Exploratory innovation has a significant U-shaped relationship with innovation performance, i.e., as the level of exploratory innovation increases, the innovation performance of elder employees first decreases and then increases. There is a significant inverted U-shaped relationship between exploitative innovation and innovation performance, i.e., as the level of exploitative innovation increases, innovation performance first increases and then decreases. High-involvement work practices have a U-shaped effect on elder employees’ innovation performance through exploitative innovation. (3) Transformational leadership moderates the direct effects of high-involvement on exploratory innovation and elder employees’ innovation performance, and transformational leadership moderates the U-shaped effect of high-involvement work practices on elder employees’ innovation performance through exploratory innovation.

**Discussion:**

The conclusion is helpful for organizations to enhance elder employees’ innovation performance by enriching high-involvement work practices.

## Introduction

In an era characterized by volatility, uncertainty, complexity and ambiguity (VUCA), organizations face unprecedented challenges and opportunities. This dynamic environment requires a heightened capacity to adapt and innovate, which is essential for both survival and growth (Bennett and Lemoine, 2014). As external competition intensifies, a critical internal dynamic is emerging within organizations - the increasing prevalence of elder employees (Gordon and Arvey, 2004). Often undervalued, these individuals possess a wealth of experience and knowledge that is proving indispensable in navigating today’s complex business terrain (Jiang et al., 2021). Their deep understanding of organizational history, resilience in the midst of transformative change, and ability to mentor junior colleagues are instrumental in fostering innovation and facilitating organizational development (Jiang et al., 2021). However, the global trend toward an aging workforce is accompanied by escalating intergenerational competition (Ward et al., 2021). As Gordon and Arvey (2004) note, many organizations priorities attracting younger talent with innovative ideas and skills, often through higher salaries and positions, while overlooking the valuable expertise and experience of elder employees (Gordon and Arvey, 2004). This oversight represents a missed opportunity to harness the full innovative potential of an organization’s workforce. Effectively harnessing and managing the innovation performance of elder employees can significantly enhance an organization’s ability to innovate. How can the HR effectiveness of older employees be effectively developed and utilized so that they can participate more in innovation practices? Based on this problem, this study focuses on a key variable - the innovation performance of elder employees, and attempts to reveal the antecedent variables and mechanisms that affect the innovation performance of elder employees.

The existing literature has extensively investigated various factors that influence the innovation performance of elder employees. These studies have primarily focused on work experience or professional expertise (Schmader et al., 2008; Lamont et al., 2015), leadership support, and employee collaboration (Huang, 2019; Cao et al., 2022). However, a significant portion of this research has focused on the impact of individual and leadership factors on elder employees. Recognizing this gap, our study aims to explore the potential of organizational strategies in catalyzing and transforming the rich knowledge and experience of elder employees into innovative behaviors and performance. To this end, high-involvement work practices are introduced as a key variable for investigation. High-involvement work practices are a type of human resource management practice that emphasizes employee involvement and empowerment. Previous studies have confirmed that high-involvement work practices have a significant positive impact on employee innovation (Becker and Gerhart, 1996; Cao et al., 2022; Wang et al., 2022). Therefore, we have reason to believe that it still has a strong explanatory potential for the innovation performance of elder employees.

Unfortunately, most of the existing literatures on the relationship between high-involvement work practices and employees’ innovation performance focus on overall innovation activities (Misnah et al., 2020; Cao et al., 2022; Wang et al., 2022; Zeb et al., 2022), but fail to specifically analyze different innovation modes. This is undoubtedly not in line with the current situation, where we urgently need to use different innovation modes to promote the development of enterprise innovation. To achieve the construction of core competitiveness and long-term sustainable development, enterprises need to combine conservatism and breakthrough in innovation activities, that is, to realize exploratory innovation based on exploitative innovation (Limaj and Bernroider, 2019). For employees, the two innovation modes, exploratory and exploitative, show significant differences in terms of implementation difficulty, time, risk, reward and motivation (Cao et al., 2022). Specifically, exploratory innovation tends to be more difficult to implement, takes longer, involves higher risks, but potentially offers greater rewards. Conversely, exploitative innovation is generally easier to implement, takes less time, carries lower risks, but offers lower rewards (Klotz et al., 2018; Tortia et al., 2022). Therefore, the impact of these two modes of employee innovation on the innovation performance of elder employees may be complex and non-linear. In light of this, it is necessary to explore the internal mechanism by which high-involvement work practices influence the innovation performance of elder employees through dual innovation.

In addition to work practices, leadership is also an important variable influencing employee innovation in organizations (Hughes et al., 2018). As leaders of organizational activities, leaders play an important role in guiding and supporting employees’ innovative behaviors (Chen et al., 2022). Traditionally, transformational leadership has been viewed as having a positive impact on employee innovation, largely due to its emphasis on inspiration, intellectual stimulation and individualized consideration (Jansen et al., 2006). However, the interaction between transformational leadership and high-involvement work practices may have unintended negative effects on the innovative behavior and performance of elder employees. The inspirational and challenging nature of transformational leadership, when combined with the high degree of autonomy required in high-involvement work practices, could impose additional stress on older employees, potentially hindering their innovative capabilities (Wang and Howell, 2010). What is more, the future-oriented and big-picture goals emphasized by transformational leaders may not align with the immediate, practical concerns of elder employees in high-involvement settings, leading to a mismatch of expectations and goals (De Clercq and Mustafa, 2023). Given these potential problems, it is essential to examine the moderating role of transformational leadership in the context of high-involvement work practices, particularly with regard to the innovation behavior and performance of older employees.

The innovation of this paper is as follows: (1) Examine how high-involvement work practices influence the innovation performance of older employees, thereby extending research on the antecedents of innovation performance among this population. (2) Investigate the mediating role of dual innovation behavior in the relationship between high-involvement work practices and the innovation performance of older employees. This research clarifies the non-linear effects of dual innovation as a crucial transmission mechanism. (3) By examining the moderating role of transformational leadership, a dominant leadership style, the study delineates the boundary conditions under which high-involvement work practices affect the innovation behavior and performance of older employees.

## Theoretical bases and research hypothesis

### Social exchange theory

The main idea of social exchange theory is ‘tangible exchange’ or ‘intangible exchange’ between parties and groups or organizations in order to obtain returns (Valentinov and Hajdu, 2019). From the perspective of social exchange theory, the interaction of resources between subjects can be seen as reciprocal exchange behavior (Ashforth and Mael, 1989). Employees not only perform to reciprocate the good treatment they receive from the organization, such as involvement in decision making, performance rewards and training, but also exchange resources with the organization due to the high level of trust. There are two typical logics based on this theory, namely market logic and social logic. Market logic emphasizes the characteristics of equality, certainty and predictability of returns when resources are exchanged between organizations. The market logic emphasizes that the exchange of organizational resources has the characteristics of inequality, uncertainty and difficulty in measuring the return, which highlights the importance of trust in the organization.

Social exchange theory has become a common perspective for studying the impact of high-involvement work practices on employee behavior (Konovsky and Pugh, 1994; Wayne et al., 1997; Cropanzano and Mitchell, 2005). For organizations, it is true that high-involvement work practices can positively stimulate employee creativity. However, based on this perspective, employees’ innovative behavior is still a passive response to organizational management measures and organizational atmosphere, which cannot reflect the concept cultivation and practice stimulation of high-involvement work practices for employees’ innovative activities. In this regard, Ryan and Deci (2000) proposed that self-determination theory could be used to improve and complement the active influence of high-involvement work practices on employees’ innovative behavior. According to self-determination theory, human behavior is driven by internal and external motivation (Alegría et al., 2014; Sharot and Sunstein, 2020). Specifically, an individual’s behavior is mainly driven by internal motivation, but under the action of external motivation, an individual chooses to participate in a job or activity that does not interest him or her.

Based on this theoretical logic, high-involvement work practices can increase employees’ work motivation and encourage them to take the initiative to innovate in two ways. First, giving employees more autonomy can increase their intrinsic motivation, satisfy their basic psychological needs for self-determination, and enhance their creativity. Second, organizations can provide more resources for employees to innovate, such as providing opportunities for employees to participate in training and providing innovative capabilities to match their high motivation to innovate. Therefore, based on social exchange theory, this paper explores the passive driving and subjective cultivation of high-involvement work practices on employees’ innovative behavior, in order to improve the influence mechanism of these work practices on older employees’ innovative performance, and to provide effective management methods for enterprises to enhance employees’ innovative vitality and ability level.

### High-involvement work practices and innovation performance

High-involvement work practices are a comprehensive and multifaceted system of work practices (Waseem et al., 2020) that includes five specific practice activities, including full empowerment, capacity development, information sharing, appreciation and recognition, and fairness in return. Compared to the individual work practice mode, high-involvement work practices can positively predict employee work outcomes. It emphasizes the long-term communication relationship between employees and the organization and encourages employees to actively participate in the organization’s work practices.

This study suggests that the implementation of high-involvement work practices can effectively improve the innovation performance of elder employees. Specifically, on the one hand, based on social exchange theory, when elder employees perceive the importance and concern of the organization, they will increase their work enthusiasm and creativity out of a willingness to give back to the organization (Kehoe and Wright, 2010). First, from the perspective of full empowerment of high-involvement work practices, through high-involvement work practices, elder employees can obtain sufficient work autonomy and decision-making power (Zeb et al., 2021a,b), so that elder employees can independently arrange and plan work content and make independent decisions. This allows elder employees to maximize their limited energy and work vitality in their work tasks and to fully develop their potential and creativity. Second, from the perspective of the characteristics of high-involvement work practices, through multiple modules including knowledge and skills training, job rotation and job coaching, capacity development activities can enrich the professional knowledge and skills of elder employees, encourage elder employees to acquire new technologies and skills that control current and future development, and stimulate workers to refine existing products and services. They actively seek creative solutions to complex problems (Woodman et al., 1993; Mustafa et al., 2018). Finally, the information-sharing characteristics of high-involvement work practices can help elder employees gather work-related information and strengthen the foundation of innovation for elder employees. Finally, the information-sharing characteristics of high-involvement work practices can help elder employees to gather work-related information and strengthen the basis of innovation for elder employees. On the other hand, based on self-determination theory, high-involvement work practices can satisfy elder employees’ basic psychological need for self-determination and then positively affect their creativity levels by improving their internal motivation (Shin et al., 2022). In addition, a sense of fairness directly leads to higher levels of commitment and effort, which are crucial for driving innovation (Paré and Tremblay, 2007). High-involvement work practices, characterized by their emphasis on fairness and equity, significantly enhance the innovation performance of elder employees by increasing their self-confidence, sense of control and commitment to their work. In summary, this paper proposes the following hypotheses:

*H*1: High-involvement work practices have a significant positive impact on elder employees’ innovation performance.

### The mediating effect of exploratory and exploitative innovation

Exploratory innovation is radical innovation that disrupts and reconstructs existing products or services (Benner and Tushman, 2003), often involving the updating and iteration of products and services and the creation of new markets. The implementation of exploratory innovation requires employees to have both the ability to break through innovation and the power to implement innovation, which is consistent with the way employees are trained in high-involvement work practices. Firstly, organizations with high-involvement work practices pay more attention to cultivating employees’ knowledge, skills and work experience than ordinary organizations; this helps elder employees to develop a sense of empowerment. In this environment, employees’ abilities are developed and it is easier for them to acquire work skills that promote creativity and expand their thinking and problem-solving ideas (Kehoe and Wright, 2010), which is conducive to the generation of employees’ innovative ideas. It is also conducive to encouraging employees to engage in innovative activities out of feedback to the organization (Zeb et al., 2020). Second, high-involvement work practices, particularly through their characteristic of granting full empowerment to employees (Kilroy et al., 2016), significantly promote exploratory innovation behaviors. In such organizational settings, employees are given greater autonomy over their daily work planning and decision making. This autonomy enables employees to effectively match their work challenges with their capabilities and resources, creating an optimal environment for the use of their task management skills (Jiang et al., 2012). They are more likely to venture into new areas, experiment with new ideas and take the initiative in innovative endeavors due to the trust and freedom provided by high-involvement work practices (Kilroy et al., 2020).

In conclusion, organizations that implement high-involvement work practices are conducive to employees learning more knowledge and mastering more resources, which is conducive to the cultivation of employees’ creative thinking and the development of the need for self-determination. At the same time, employees perceive full empowerment and trust from the organization and are more willing to engage in exploratory innovation to give back to the organization. On this basis, this paper proposes the following hypothesis:

*H*2: High-involvement work practices have a significant positive influence on exploratory innovation.

Exploratory innovation is when a firm deviates from existing products and markets and tries to develop new products and services for new customers or markets (Jansen et al., 2006). At the individual employee level, however, exploratory innovation involves deviating from established routines and venturing into the development of new markets (Weber et al., 2022). For elder employees, low levels of exploratory innovation may initially lead to a decline in innovation performance. This decline has been attributed to the significant investment of time and resources required in the early stages of exploratory innovation, which may not yield immediate benefits and may be particularly challenging for elder employees who may be less accustomed to rapid shifts in innovation paradigms (Chi and Lin, 2011). These initial efforts into uncharted territory often involve high levels of uncertainty and risk, which may negatively impact the short-term innovation performance of these employees (Woods et al., 2018).

However, as the level of exploratory innovation increases, elder employees gradually accumulate new knowledge, develop new competencies and begin to adopt more forward-looking and innovative approaches (Jiang et al., 2021). This development leads to an enhanced ability to identify and respond to market changes and technological advances. Over time, sustained engagement in exploratory innovation allows elder employees to break out of conventional thinking patterns and adopt more dynamic and disruptive approaches (Quintus et al., 2017). As a result, their innovation performance begins to improve, benefiting from the broader perspective and adaptability gained through sustained exploratory activity (Collins et al., 2009). In essence, while the initial stages of exploratory innovation may temporarily hamper elder employees’ innovation performance, continued and increased engagement in such activities fosters an environment conducive to long-term innovation success. On this basis, this paper proposes the following hypothesis:

*H*3: Exploratory innovation has a significant U-shaped relationship with elder employees’ innovation performance, that is, with the improvement of exploratory innovation level, elder employees’ innovation performance first decreases and then increases.

Organizations with high-involvement work practices emphasize the development of employees’ skills and cultivate their work initiative (Song et al., 2009). In such an environment, employees are more likely to be stimulated to enhance their creativity and engage in exploratory innovation behavior. When employees engage in exploratory innovation behavior for a long time, it is easier for them to form their vision of innovation and development on the existing problems and future development of the firm, which encourages them to promote new innovation and improve the innovation performance of older employees in the firm (Woods et al., 2018). At the same time, combined with hypothesis H2 and hypothesis H3, high-involvement work practices positively affect exploratory innovation, and exploratory innovation has a U-shaped influence on innovation performance. Therefore, it can be concluded that exploratory innovation plays a mediating role in the relationship between high-involvement work practices and innovation performance. Based on this, the following hypothesis is proposed in this paper:

*H*4: High-involvement work practices have U-shaped influence on elder employees’ innovation performance through exploratory innovation.

Exploitative innovation is a progressive innovation to improve existing products and services, which often involves upgrading existing products and services, technological improvement, cost reduction and market expansion (Chen et al., 2014). As a comprehensive practice, high-involvement work practices can stimulate employees’ exploitative innovation behavior from many aspects. First, creating a good atmosphere for innovation is an important way to motivate employees to innovate (García-Morales et al., 2012). Organizations with high-involvement can improve employees’ perceptions of an innovative work environment by forming an organizational innovation atmosphere, so that employees can gradually increase their intrinsic motivation to innovate in a good organizational innovation atmosphere, stimulate their enthusiasm for innovation, establish and form an innovation system of self-management and incentives, and improve their exploitative innovation efficiency. Second, employees with high-involvement in work organization have greater job autonomy, which makes them more flexible and free in carrying out exploitative innovation activities, which not only helps them to have the expectation of giving back to the organization, but also increases their need for self-determination, thus encouraging employees to give full play to their creativity, thinking and problem-solving abilities (Woodman et al., 1993). Finally, the information sharing provided by high-involvement work practices can help employees more efficiently obtain work information conducive to exploitative innovation activities, and multiple information channels and abundant information resources can lay a solid foundation for employees to carry out exploitative innovation (Chen et al., 2021). Based on this, this paper proposes the following hypothesis:

*H*5: High-involvement work practices have a significant positive impact on exploitative innovation.

Exploitative innovation at the employee level involves refining, iterating and improving existing products and services (Jansen et al., 2006). Initially, a moderate level of exploitative innovation can lead to an increase in the innovation performance of elder employees. Older employees, with their extensive experience and familiarity with current systems and processes (Quintus et al., 2017), are particularly adept at making these incremental changes. They are able to adapt quickly to market changes and make timely adjustments to products and services, thereby maintaining organizational stability and improving their own innovation performance.

However, as the level of exploitative innovation intensifies beyond a certain point, its positive impact on the innovation performance of elder employees begins to decline. This decline is attributed to the inherent limitations of focusing too heavily on refining existing products, which can lead to stagnation in creativity and a lack of fresh, transformative ideas (Limaj and Bernroider, 2019). Older employees may feel constrained by the repetitive nature of continuous incremental improvement (Jiang et al., 2021), leading to reduced motivation and engagement in innovation activities. Consequently, while initial levels of exploitative innovation may enhance older employees’ innovation performance, an excessive focus on this form of innovation may ultimately hinder their long-term innovation capabilities and contributions. On this basis, this paper proposes the following hypothesis:

*H*6: Exploitative innovation has a significant inverted U-shaped relationship with elder employees’ innovation performance, that is, with the improvement of the level of exploitative innovation, elder employees’ innovation performance first increases and then decreases.

Based on the above analysis, high-involvement work practices are conducive to the formation of organizational innovation atmosphere and information sharing mode to promote exploitative innovation. To some extent, exploitative innovation can have a positive impact on organizations to identify innovation opportunities, carry out innovation activities effectively and improve the innovation performance of older employees. However, as the level of exploitative innovation increases further, it may limit employees’ innovative thinking and dampen their enthusiasm for innovation, which subsequently leads to a decline in older employees’ innovation performance (Limaj and Bernroider, 2019). Combined with hypotheses H5 and H6, high-involvement work practices positively influence exploitative innovation, and exploitative innovation has an inverted U-shaped influence on older employees’ innovation performance. In conclusion, exploitative innovation plays a mediating role between high-involvement work practices and elder employees’ innovation performance. Therefore, the following hypothesis is proposed:

*H*7: High-involvement work practices have an inverted U-shaped effect on elder employees’ innovation performance through exploitative innovation.

### The moderating effect of transformational leadership

Transformational leadership is a leadership style that motivates and inspires followers through a shared vision, intellectual stimulation, individualized consideration and by being a role model (García-Morales et al., 2012; Fernet et al., 2015; Arnold, 2017; Zeb et al., 2020). Most studies consider transformational leadership as a leadership style that focuses on improving the intrinsic motivation of subordinates and emphasizes influencing subordinates through leadership charm, charisma, intellectual stimulation and personalized care (Wright and Pandey, 2009). However, transformational leadership can also have a negative impact on the innovation behavior of older employees. By frequently challenging non-conformist ideas, transformational leaders may inadvertently make subordinates overly dependent on their direction (Jiang and Chen, 2021), thus discouraging the independent and creative thinking essential for exploratory innovation. Furthermore, the pressures of transformational leadership can sometimes be overwhelming, reducing individual creative output, particularly in high-involvement work settings that require employee autonomy (Men et al., 2020). Research such as Chen et al. (2022) shows that although transformational leadership can enhance group innovation, it can have a negative impact on individual innovation (Chen et al., 2022). This suggests that in environments with high-involvement practices, strong transformational leadership may actually reduce the effectiveness of these practices in promoting individual exploratory innovation.

Conversely, in environments where transformational leadership is less pronounced, the positive effects of high-involvement work practices on exploratory innovation become more evident. In environments with lower levels of transformational leadership, employees are less influenced by a leader’s vision and direction, giving them more freedom to pursue novel ideas and approaches independently. This autonomy is a key component of high-involvement work practices, which emphasize employee empowerment and participative decision making (Men et al., 2020). Without the overshadowing influence of a strong transformational leader, employees can explore and experiment more freely, reaping the full benefits of high-involvement practices. In such contexts, these practices effectively promote a culture of exploration and creativity, as employees feel more confident and able to take the initiative in innovative endeavors (Chen et al., 2022). Therefore, the predictive effect of high-involvement work practices on exploratory innovation is likely to be stronger in scenarios characterized by lower levels of transformational leadership, allowing employees to fully engage in and benefit from the empowering and inclusive nature of these practices. Based on this, transformational leadership is introduced as a moderating variable affecting the relationship between high-involvement work practices and exploratory innovation, and the following hypotheses are proposed:

*H*8: Transformational leadership moderates the influence of high-involvement work practices on exploratory innovation, that is, the lower the level of transformational leadership, the stronger the predictive effect of high-involvement work practices on exploratory innovation.

Similarly, transformational leadership may negatively moderate exploitative innovation. In environments where transformational leadership is less pronounced, employees may rely more on the structured, empowering aspects of high-involvement work practices that are critical for incremental and process-oriented innovation (Tipu et al., 2012). Such an environment allows for greater autonomy and decision making at the individual and team level, fostering an atmosphere in which exploitative innovation can flourish. In contrast, higher levels of transformational leadership, while beneficial in many contexts, may overshadow the process-oriented, systematic approach of high-involvement work practices, potentially reducing their impact on exploitative innovation. Transformational leaders, with their emphasis on visionary goals and radical change, may inadvertently divert focus and resources away from the incremental improvements that characterize exploitative innovation (Jansen et al., 2006). Therefore, it is proposed that the positive influence of high-involvement work practices on exploitative innovation may be more pronounced in settings with lower levels of transformational leadership, as these practices may more directly influence and shape the innovative efforts of the workforce. Based on this, transformational leadership is introduced as a moderating variable affecting the relationship between high-involvement work practices and exploitative innovation, and the following hypotheses are proposed:

*H*9: Transformational leadership moderates the influence of high-involvement work practices on exploitative innovation, that is, the lower the level of transformational leadership, the stronger the predictive effect of high-involvement work practices on exploitative innovation.

High-involvement work practices typically promote participatory decision making, autonomy in work roles and a sense of ownership of work processes, which may be particularly effective in enhancing elder employees’ innovation performance (Hauff et al., 2022). In contexts where transformational leadership is less dominant, elder employees may experience more autonomy and empowerment, key tenets of high-involvement work practices. Without the strong influence of transformational leadership, which often directs focus toward a visionary goal, elder employees may have more opportunities to use their extensive experience and knowledge in a self-directed manner, leading to increased innovative performance (Arnold, 2017). On the other hand, in environments with high levels of transformational leadership, the directive and charismatic nature of such leadership may overshadow the autonomy and empowerment provided by high-involvement work practices (Wang et al., 2022). This could potentially limit the ability of elder employees to fully engage in innovative behaviors, as their actions and decisions may become more aligned with the transformational leader’s vision rather than their own creative and experiential insights (Klonek et al., 2023). This implies that the lower the presence of transformational leadership, the more pronounced the positive effects of high-involvement work practices on the innovation performance of elder employees. Based on this, this paper introduces transformational leadership as a moderator that influences the relationship between high engagement in work practices and elder employees’ innovation performance, and proposes the following hypotheses:

*H*10: Transformational leadership moderates the influence of high-involvement work practices on elder employees’ innovation performance, that is, the lower the level of transformational leadership, the stronger the predictive effect of high-involvement work practices on elder employees’ innovation performance.

### Moderated mediation

It is hypothesized that H4 and H8 together form the moderated mediation effect, i.e., exploratory innovation mediates the influence of high-involvement work practices on elder employees’ innovation performance. By moderating the positive effect of high-involvement work practices on exploratory innovation, transformational leadership moderates the effect of high-involvement work practices on elder employees’ innovation performance through exploratory innovation. H7 and H9 are also hypothesized to constitute the moderated mediation effect, i.e., exploitative innovation mediates the influence of high-involvement work practices on elder workers’ innovation performance. Transformational leadership moderates the positive effect of high-involvement work practices on exploitative innovation, and then moderates the effect of high-involvement work practices on elder workers’ innovation performance. Therefore, the following hypothesis is proposed:

*H*11: Transformational leadership moderates the inverted U-shaped influence of high-involvement work practices on elder employees’ innovation performance through exploratory innovation.

*H*12: Transformational leadership moderates the U-shaped influence of high-involvement work practices on elder employees’ innovation performance through exploitative innovation.

**Figure 1:**
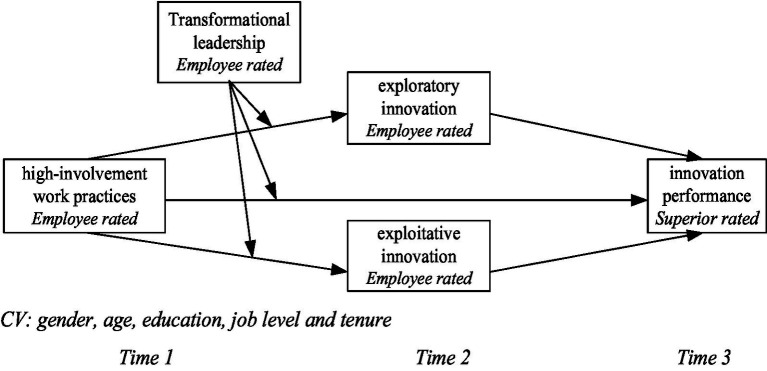
Research theoretical model.

To sum up, the research model in this paper is shown in Figure 1.

## Research design

### Data

This study used a questionnaire survey method to collect data. Data was collected from elder employees in 15 technology companies in eastern China, including the software development industry, telecommunications industry, steel manufacturing industry, etc. Elder employees in these industries face a lack of innovation. Therefore, we investigate whether the implementation of high-involvement work practices in these industries can contribute to the improvement of innovation performance. Most studies define elder employees as those over the age of 35 (Collins et al., 2009), and this is the criterion used in this study. Initially, the company representative was contacted to explain the purpose of the study and to ask for volunteers to participate in the survey. The survey was conducted through a combination of online questionnaires and on-site interviews. Links to the questionnaires were distributed to individual employees. Paper questionnaires were distributed to survey participants for those who did not wish to complete them online. Each respondent was coded before completing the questionnaire and the questionnaires were matched by coding after they were returned. In order to ensure the authenticity and validity of the data as much as possible, respondents were assured that their answers would be anonymous and that a certain payment (40 yuan) would be paid to participants at the end of each survey.

To reduce potential common methodological biases (Podsakoff et al., 2003), the study was conducted in three phases. Each phase was separated by 3 weeks, as too long a time gap may obscure existing relationships and conversely, too short a time interval may inflate the relationship between variables due to memory effects (Babalola et al., 2019). In the first phase, employees reported on high-involvement work practices, transformational leadership and demographics. A total of 400 questionnaires were sent out and 367 were collected, with a return rate of 91.75%. Three weeks later, in the second phase, 367 employees who participated in the first phase of the survey were given questionnaires and employees reported on exploratory innovation, exploratory innovation, work pressure and demographic characteristics. 336 questionnaires were collected, giving a response rate of 91.55%. Three weeks later, in the third stage, questionnaires were distributed to the supervisors of the employees who had participated in the second stage of the survey. The supervisors reported on the employees’ innovation performance and demographic characteristics, and 305 questionnaires were collected, giving a response rate of 90.77%. Of the 305 complete responses, a total of 278 valid questionnaires were obtained, excluding those with inconsistent demographic variables, invalid, regular and excessive missing data. We used the methods of Armstrong and Overton (1977) (Armstrong) to assess potential non-response bias. The chi-squared test and independent samples t-test were used to compare the first 85 respondents with the second 85 respondents using demographic variables such as age and gender. The results showed that there was no significant difference between the two groups (*p* > 0.05).

### Measurement of variables

All scales used a seven-point Likert scale, ranging from 1 (strongly disagree) to 7 (strongly agree). All scales used have been shown to have good psychometric properties.

High-involvement work practices: High-involvement work practices is a scale developed by Jiang et al. (2021), consisting of 29 questions from seven dimensions, including personnel, training and development, performance management and evaluation, compensation and benefits, job design, participation and autonomy, and information sharing, the internal consistency coefficients were 0.885, 0.800, 0.797, 0.921, 0.873, 0.847, 0.855, respectively. It is translated into Chinese by following the traditional translation back procedure (Bhawuk and Brislin, 2001; Kehoe and Wright, 2010; White et al., 2013). Respondents rated how often they experienced each condition on a scale of 1 (strongly disagree) to 7 (strongly agree). Items included “My workplace is able to hire people with the right skills.” And “I’ve been given a real opportunity to improve my skills in the organization.” The internal consistency coefficient of high-order and high-participation work practices was 0.971. The second-order seven-factor confirmatory factor analysis was carried out on the high participation work practices scale. The results were as follows: χ^2^/*df* = 2.570, IFI = 0.919, TLI = 0.907, CFI = 0.918, RMSEA = 0.075. The model fitting effect was good. In line with common practice in this field, the seven dimensions are examined as a whole. Research has focused on the synergistic effects of high-involvement work practices (Vandenberg et al., 1999) and therefore, the internal consistency coefficient of the scale was 0.971.

High-involvement work practices: High-involvement work practices is a scale developed by Jiang et al. (2021). The scale consists of 29 questions from seven dimensions, including human resources, training and development, performance management and evaluation, compensation and benefits, job design, participation and autonomy, and information sharing, with internal consistency coefficients of 0.885, 0.800, 0.797, 0.921, 0.873, 0.847, and 0.855, respectively. It was translated into Chinese using the traditional translation-back procedure (Bhawuk and Brislin, 2001; Kehoe and Wright, 2010; White et al., 2013). Respondents rated how often they experienced each condition on a scale of 1 (strongly disagree) to 7 (strongly agree). Items included ‘My workplace is able to recruit people with the right skills’ and ‘I have been given a real opportunity to develop my skills in the organization’. The internal consistency coefficient of the high order and high participation work practices was 0.971. The seven-factor second-order confirmatory factor analysis was carried out on the high participation work practices scale. The results were as follows: χ^2^/*df* = 2.570, IFI = 0.919, TLI = 0.907, CFI = 0.918, RMSEA = 0.075. The model fit was good. In line with common practice in the field, the seven dimensions are examined as a whole. Research has focused on the synergistic effects of high-involvement work practices (Vandenberg et al., 1999) and therefore the internal consistency coefficient of the scale was 0.971.

Exploitative innovation and exploratory innovation: Exploitative innovation and exploratory innovation is measured by the 8-item scale (Benner and Tushman, 2003). Respondents rated how often they experienced each condition on a scale of 1 (strongly disagree) to 7 (strongly agree). Exploitative innovation includes “often develop new market segments without relevant marketing experience” and “often adopt business strategies not used by other companies in the same industry,” and the internal consistency coefficient is 0.918. Exploratory innovation includes “striving to improve the applicability of existing technologies/skills in multiple related business fields” and “frequently using existing technologies/skills to increase the diversity of functions and products/services,” with an internal consistency coefficient of 0.937.

Transformational leadership: The 8-item scale of Li and Shi (2008; Guay and Choi, 2015) was used to measure transformational leadership. Respondents rated how often they experienced each condition on a scale of 1 (strongly disagree) to 7 (strongly agree). Items included “My boss can share the difficulties of my employees.” And” My leader can keep employees informed about the future of the department.” The internal consistency coefficient of the scale is 0.954.

Innovation performance: The 10-item scale of Janssen and Yperen (2004) was used to measure innovation performance. Respondents (superiors) rated how often the employee experienced each condition on a scale of 1 (strongly disagree) to 7 (strongly agree). Items included “At work, He or She often challenge problems that have not been solved.” And” He or She is excited to come up with new ideas to improve things.” The internal consistency coefficient of this scale is 0.976.

Control variables: Referring to existing research practice (Gu et al., 2015; Wang et al., 2022), gender (0 = female, 1 = male), age (1 = 35–45, 3 = 56 and above), education (1 = high school education or below, 4 = master’s degree or above), job level (1 = general employee, 4 = senior managers) and tenure (1 = less than 3 years, 4 = more than 10 years) were included in the study as control variables.

## Results and analysis

### Descriptive analysis

SPSS 24 and Mplus 8.0 software are used for data analysis in this paper. The mean, standard deviation and correlation of all studied variables was listed in Table 1. As shown in Table 1, the correlation of the study variables is in the expected direction, and the internal consistency of the study variables is within the acceptable range. High-involvement work practices was positively correlated with exploitative innovation (*r* = 0.713, *p* < 0.01), exploratory innovation (*r* = 0.340, *p* < 0.01), and innovation performance (*r* = 0.547, *p* < 0.01). In addition, exploitative innovation and exploratory innovation were positively correlated with elder employees’ innovation performance (*r* = 0.608, *p* < 0.01; *r* = 0.493, *p* < 0.01).

**Table 1:** Matrix of mean value, variance and correlation coefficient.

	Mean value	Standard deviation	1	2	3	4	5	6	7	8	9	10
1. Gender	0.670	0.514	0.264	0.093	0.010	−0.007	0.129	−0.085	−0.068	0.093	−0.054	−0.012
2. Age	2.370	0.726	0.250^**^	0.527	−0.033	0.125	0.544	0.068	0.093	0.224	0.196	0.032
3. Education	2.440	0.745	0.027	−0.061	0.555	0.107	−0.241	−0.045	−0.028	0.197	−0.066	−0.001
4. Job level	1.250	0.593	−0.024	0.291^**^	0.241^**^	0.352	0.176	0.191	0.181	0.230	0.217	0.247
5. Tenure	3.380	1.202	0.208^**^	0.624^**^	−0.270^**^	0.247^**^	1.444	0.062	0.182	0.354	0.374	0.056
6. HIWPS	4.925	0.983	−0.169^**^	0.095	−0.061	0.328^**^	0.052	(0.755)	0.926	0.405	0.816	0.674
7. TL	4.990	1.079	−0.123^*^	0.119^*^	−0.035	0.283^**^	0.140^*^	0.877^**^	(0.871)	0.450	0.866	0.692
8. EXR	3.875	1.212	0.149^*^	0.254^**^	0.218^**^	0.320^**^	0.243^**^	0.340^**^	0.344^**^	(0.897)	0.859	0.673
9. EXI	4.867	1.167	−0.090	0.231^**^	−0.076	0.313^**^	0.266^**^	0.713^**^	0.687^**^	0.608^**^	(0.918)	0.722
10. IP	4.694	1.252	−0.019	0.035	−0.001	0.332^**^	0.037	0.547^**^	0.512^**^	0.443^**^	0.493^**^	(0.908)

*p < 0.05; **p < 0.01. The lower left matrix is the correlation coefficient, the upper right matrix is the covariance matrix, and inside the diagonal parentheses is the square root of AVE. HIWPS, high-involvement work practices; TL, transformational leadership; EXR, exploratory innovation; EXI, exploitative innovation; IP, innovation performance.

### Measurement model

The convergence validity and discrimination validity of the measurement model were tested. The results of confirmatory factor analysis (CFA) show that the five-factor model (i.e., high-involvement work practices, exploratory innovation, exploitative innovation, innovation performance, transformational leadership) has a good fit: χ^2^ (df = 1,338) =2735.984, *p* < 0.01, RMSEA = 0.062, CFI = 0.910, TLI = 0.904, IFI = 0.911 (Table 2), and the load of each factor had statistical significance (*p* < 0.01). The results of model comparisons further indicate that the hypothetical five-factor measurement model has a better fit to the data than any alternative four-factor model (i.e., combining any two of the five factors).

**Table 2:** Results of confirmatory factor analysis.

Model	χ^2^	df	χ^2^/df	RMSEA	IFI	TLI	CFI
Single factor	6336.387	1,355	4.676	0.115	0.682	0.662	0.681
Double factor	5443.227	1,347	4.041	0.105	0.739	0.721	0.737
Three factor	5085.245	1,345	3.781	0.100	0.761	0.745	0.760
Four factor	4187.304	1,342	3.120	0.088	0.818	0.805	0.817
Five factor: theoretical model	2735.984	1,338	2.045	0.062	0.911	0.904	0.910
Six factor	2718.618	1,336	2.035	0.061	0.912	0.905	0.911

Single factor, all variables; Double factor, HIWPS, TL + EXR + EXI + IP; Three factors, HIWPS, TL + EXR + EXI, IP; Four factors, HIWPS, TL, EXR + EXI, IP; Six factors, five factors plus CMV.

### Common method bias

Although this study uses data from three stages, all the data are self-reported by employees, so there are inevitable endogeneity problems. To test endogeneity, Harman’s single factor test and ULMC (Unmeasured Latent Method Construct) were used to test the presence of common method bias (Podsakoff et al., 2003). As shown in Table 2, the single factor model was not suitable [χ^2^(1355) = 6336.387, *p* < 0.01; CFI = 0.681, TLI = 0.662 and RMSEA = 0.115], while the five-factor model met the requirements [χ^2^(1338) = 2735.984, *p* < 0.01, CFI = 0.911, TLI = 0.905, and RMSEA = 0.061]. The χ^2^ comparison showed that the single factor model was significantly worse than the five factor model.

At the same time, referring to the practice of Liang et al. (2007), the method of ULMC method factors was used to test the influence of common method bias factors. By loading all the observation indexes of five theoretical variables, a latent variable CMV was constructed, and a six-factor model including five theoretical variables and CMV was established. The results showed that the six-factor model [χ^2^(1336) = 2718.618, *p* < 0.01, CFI = 0.911, TLI = 0.905, RMSEA =0.061] had no significant improvement compared with the theoretical model (five-factor model; Table 2). Based on the above judgments, the influence of common method bias in this study is not significant.

## Hypothesis testing

### Main effect

Regression analysis was conducted for each variable, and the results were shown in Table 3. For the sake of presentation, the results are described in the order of the hypotheses. As can be seen from model 6, the effect of high-involvement work practices on elder employees’ innovation performance is not significant (*b* = 0.069, *p* > 0.05), and hypothesis H1 is not supported. As can be seen from model 3, the effect of high-involvement work practices on exploratory innovation is not significant (*b* = 0.267, *p* > 0.05), and hypothesis H2 is not supported. As can be seen from model 6, exploratory innovation has a significant U-shaped relationship with elder employees’ innovation performance (*b* = 0.101, *p* < 0.05), that is, with the increase of exploratory innovation level, elder employees’ innovation performance first decreases and then increases. Hypothesis H3 is supported. From model 1, it can be seen that high-involvement work practices has a significant impact on exploitative innovation (*b* = 0.607, *p* < 0.01), and hypothesis H5 is supported. From model 6, it can be seen that exploitative innovation has a significant inverted U-shaped relationship with elder employees’ innovation performance (*b* = −0.161, *p* < 0.01), that is, with the increase of exploratory innovation level, innovation performance first increases and then decreases. Hypothesis H6 is supported.

**Table 3:** Results of regression analysis.

Variables	EXI	EXI^2^	EXR	EXR^2^	IP	IP
	Model 1	Model 2	Model 3	Model 4	Model 5	Model 6
constant	4.026^***^	15.690^***^	1.458^***^	−2.086	4.275^***^	1.984
gender	−0.079	−0.819	0.296^*^	2.403^*^	0.071	0.059
age	0.088	1.086	0.120	1.139	−0.079	−0.057
education	0.039	0.507	0.505^***^	3.701^***^	−0.104	−0.047
job level	0.040	0.656	0.184	2.050^*^	0.464^***^	0.449^***^
tenure	0.174^***^	1.545^**^	0.188^*^	1.185^*^	−0.152^*^	−0.139^*^
HIWPS	0.607^***^	4.696^***^	0.267	1.983	0.234	0.069
EXI					0.012	1.555^***^
EXR					0.245^***^	−0.568
EXI^2^						−0.161^***^
EXR^2^						0.101^*^
TL	0.200^*^	2.676^**^	0.102	0.670	0.206	0.344^***^
TL × HIWPS	−0.058	−0.012	−0.183^***^	−1.520^***^	−0.285^***^	−0.193^***^
R^2^	0.573	0.544	0.323	0.315	0.479	0.509
F	45.092^***^	40.114^***^	16.025^***^	15.440^***^	24.563^***^	22.873^***^

*p < 0.05, **p < 0.01, ***p < 0.001.

### Test of mediating effect

The Bootstrap sampling number was set to 5,000 and the confidence level of the confidence interval was set to 95%. The result path coefficient was shown in Table 4. The results show that high-involvement work practices has no significant effect on quadratic terms of exploratory innovation (*b* = 1.983, *p* > 0.05), high-involvement work practices has significant effect on quadratic terms of exploitative innovation (*b* = 4.696, *p* < 0.000), and exploratory innovation has significant effect on elder employees’ innovation performance (*b* = 0.101, *p* < 0.05). Exploitative innovation had a significant effect on elder employees’ innovation performance (*b* = −0.161, *p* < 0.000). High-involvement work practices has no significant influence on U-shaped innovation performance through exploratory innovation, confidence interval is [−0.037, 0.623], including 0, and hypothesis H4 is not supported. The high-involvement work practices have a significant inverted U-shaped effect on elder employees’ innovation performance through exploitative innovation. The confidence interval is [−1.488, −0.288], excluding 0 and hypothesis H7 is verified.

**Table 4:** Indirect effects of high-involvement work practices on elder employees’ innovation performance.

Path	Coefficient	Standard deviation	*p* value	95% confidence interval
HIWPS→EXR^2^	1.983	1.106	0.074	[−0.195, 4.160]
EXR^2^ → IP	0.101	0.040	0.012	[0.023, 0.180]
HIWPS→EXI^2^	4.696	1.032	0.000	[2.664, 6.728]
EXI^2^ → IP	−0.161	0.042	0.000	[−0.244, −0.078]
HIWPS→EXR^2^ → IP	−0.154	0.072		[−0.037, 0.623]
HIWPS→EXI^2^ → IP	0.002	0.062		[−1.488, −0.288]

### Test of moderation effect

Model 3 in Table 3 shows that transformational leadership has a significant moderating coefficient (*b* = −0.183, *p* < 0.05) on the influence of high-involvement work practices on exploratory innovation, that is, under low level transformational leadership, high-involvement work practices has a strong influence on exploratory innovation. On the contrary, under high level transformational leadership, the positive effect of high-involvement work practices on exploratory innovation is weak, and hypothesis H8 is supported.

According to Model 1 in Table 3, the adjustment coefficient of transformational leadership on the influence of high-involvement work practices on exploitative innovation is not significant (*b* = −0.058, *p* > 0.05), that is, the prediction effect of high-involvement work practices level on exploitative innovation will not be affected by the level of transformational leadership, and hypothesis H9 is not supported.

Model 6 in Table 3 shows that transformational leadership moderates the impact of high-involvement work practices on elder employees’ innovation performance (*b* = −0.193, *p* < 0.05), that is, the lower the level of transformational leadership, the stronger the predictive effect of high-involvement work practices on elder employees’ innovation performance, and hypothesis H10 is supported.

**Figure 2:**
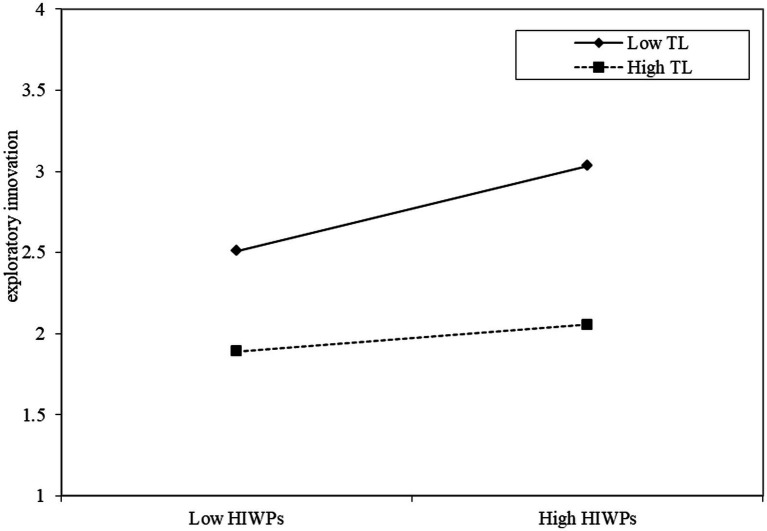
Moderating effects of transformational leadership.

**Figure 3:**
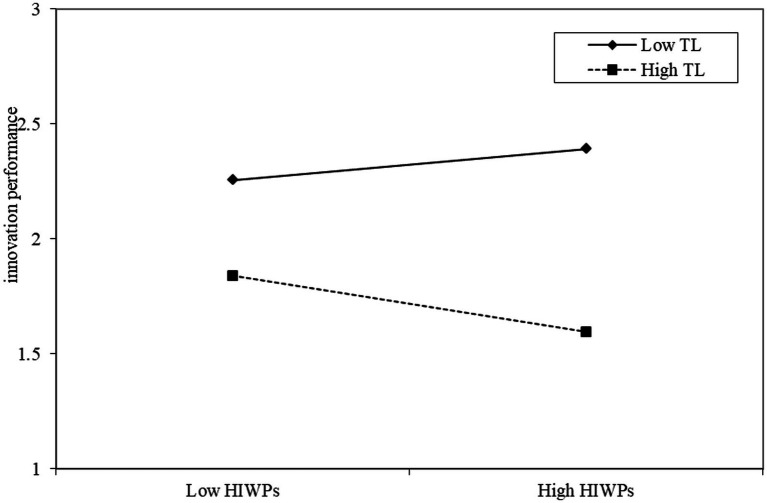
Moderating effects of transformational leadership.

To further explain the moderating effects of transformational leadership, simple slope estimates were performed (Figures 2, 3). As shown in Figure 2, under low-level transformational leadership, the influence of high-involvement work practices on exploratory innovation is stronger; on the contrary, under high-level transformational leadership, the influence of high-involvement work practices on exploratory innovation is weaker. Therefore, transformational leadership weakens the predictive effect of high-involvement work practices on exploratory innovation to some extent. As shown in Figure 3, under low-level transformational leadership, high-involvement work practices positively influences elder employees’ innovation performance, while under high-level transformational leadership; high-involvement work practices negatively influence elder employees’ innovation performance. Therefore, transformational leadership moderates the influence of high-involvement work practices on elder employees’ innovation performance.

### Test of moderated mediation

**Figure 4:**
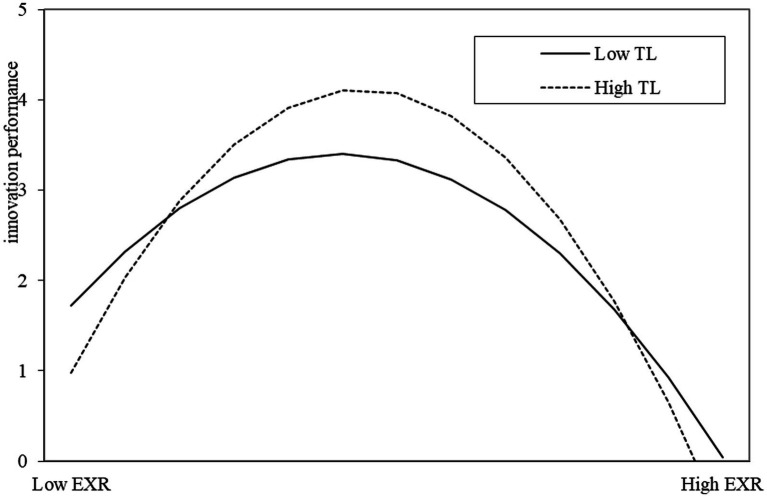
Inverted U-shaped moderating effect of transformational leadership.

Table 5 shows the indirect effect of high-involvement work practices on elder employees’ innovation performance under different levels of transformational leadership. If the 95% confidence interval does not include 0, the indirect effect can be judged to be significant. The results show that through exploratory innovation, the indirect effect of high-involvement work practices on elder employees’ innovation performance is not significant (confidence interval [−0.037, 0.623]). Through exploitative innovation, the indirect curve effect of high-involvement work practices on elder employees’ innovation performance is significant (confidence interval [−1.488, −0.288]). It can be concluded that transformational leadership moderates the high-involvement work practices through the inverted U-shaped influence of exploratory innovation on elder employees’ innovation performance (confidence interval: [−0.328, −0.040]). Meanwhile, Figure 4 also shows the moderating effect of transformational leadership on the inverted U-shaped influence, and hypothesis H11 is supported. The U-shaped effect of exploitative innovation on elder employees’ innovation performance (confidence interval: [−0.110, 0.142]) of transformational leadership in regulating high-involvement work practices is not supported, and hypothesis H12 is not supported.

**Table 5:** Indirect effects of high-involvement work practices on elder employees’ innovation performance.

Indirect effect	Innovation performance	Indirect effect	Innovation performance
Exploratory innovation		Exploitative innovation	
Average level of indirect effects	[−0.548, 0.011]	Average level of indirect effects	[0.414, 1.796]
High level	[−0.403, 0.166]	High level	[0.277, 1.671]
Low level	[−0.717, −0.032]	Low level	[0.470, 1.833]
Exploratory innovation^2^		Exploitative innovation^2^	
Average level of indirect effects	[−0.037, 0.623]	Average level of indirect effects	[−1.488, −0.288]
High level	[−0.311, 0.437]	High level	[−1.568, −0.227]
Low level	[0.087, 0.839]	Low level	[−1.432, −0.318]

The meaning of the number 2 is square.

## Conclusion

**Figure 5:**
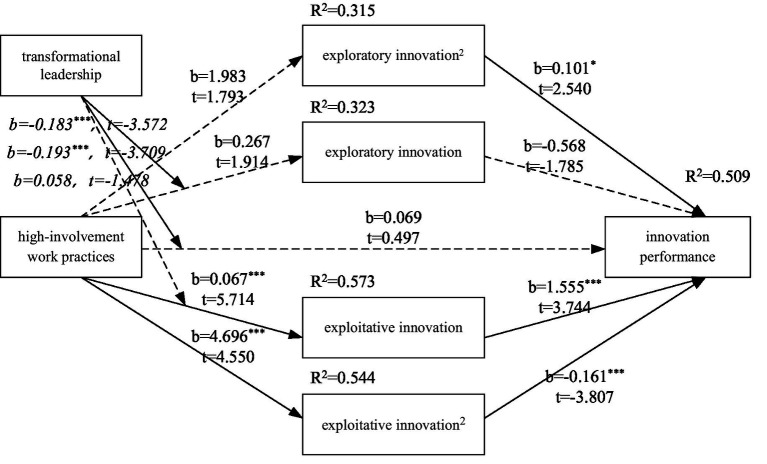
Research conclusion.

Based on social exchange theory, 278 valid samples from three time points were used to investigate the influence of high-involvement work practices on older employees’ innovation performance, as well as the non-linear effects of exploratory innovation and exploitative innovation on older employees’ innovation performance (see Figure 5). The results show that: First, high-involvement work practices do not have a significant effect on elder employees’ innovation performance. Second, high-involvement work practices have no significant effect on exploratory innovation; exploratory innovation has a significant U-shaped effect on older employees’ innovation performance, i.e., as the level of exploratory innovation increases, older employees’ innovation performance first decreases and then increases. High-involvement work practices have no significant effect on the innovation performance of older employees through exploratory innovation; high-involvement work practices have a significant positive effect on exploitative innovation; exploitative innovation has a significant inverted U-shaped effect on the innovation performance of older employees, i.e., as the level of exploitative innovation increases, the innovation performance of older employees first increases and then decreases. High-involvement work practices have an inverted U-shaped effect on innovation performance through exploitative innovation. Third, transformational leadership moderates the influence of high-involvement work practices on exploratory innovation, that is, the lower the level of transformational leadership, the stronger the predictive effect of high-involvement work practices on exploratory innovation; transformational leadership has no significant effect on the process of exploitative innovation in high-involvement work practices; transformational leadership moderates the influence of high-involvement work practices on older employees’ innovation performance, that is, the lower the level of transformational leadership, the stronger the predictive effect of high-involvement work practices on older employees’ innovation performance. Fourth, transformational leadership moderates the U-shaped influence of high-involvement work practices on older employees’ innovation performance through exploratory innovation; the effect of transformational leadership on older employees’ innovation performance through exploitative innovation in high-involvement work practices is not significant.

### Theoretical implications

First, the main contribution of this research lies in its in-depth examination of how high-involvement work practices influence the innovation performance of older employees, thereby broadening the scope of research on the antecedents of innovation performance within this specific demographic group. Previous research in this area has primarily focused on the influence of individual (e.g., work experience and expertise) and leadership factors (e.g., managerial support) on elder employees’ innovation performance (Jiang et al., 2021), often overlooking the important role that organizational practices play in shaping these outcomes. This research fills this gap by focusing on high-involvement work practices as a critical organizational factor. By exploring the ways in which these practices influence elder employees, this study provides a comprehensive understanding of the pathways of influence and the boundary conditions under which high-involvement work practices operate. This research highlights the importance of organizational practices in nurturing and capitalizing on the unique skills and experiences of elder employees.

Second, this study examines the mediating role of dual innovation - exploratory and exploitative innovation - in the relationship between high-involvement work practices and elder employees’ innovation performance. Previous studies exploring the mechanisms of innovation performance formation often treat innovation as a singular concept (Mustafa et al., 2018; Rehman et al., 2019; De Clercq and Mustafa, 2023). However, exploratory innovation, characterized by experimentation and venturing into new territories, and exploitative innovation, which focuses on refining and improving existing processes and products (Martínez-Del-Río et al., 2012), differ significantly in both approach and impact on innovation performance. This study recognizes and addresses these differences by moving away from the traditional linear research approach (Camps and Luna-Arocas, 2009; Prieto and Pilar Pérez Santana, 2012; Kilroy et al., 2020; Misnah et al., 2020b). It clarifies the non-linear effects of dual innovation, thereby establishing a new link between high-involvement work practices and innovation performance, and highlights dual innovation as a critical transmission mechanism.

Finally, this study finds that transformational leadership moderates the influence of high-involvement work practices on exploratory innovation and innovation performance. Previous research has extensively documented that transformational leadership generally has a positive influence on employee behavior and organizational performance (Laursen and Salter, 2006). Such studies have highlighted how transformational leaders, through their inspirational vision, intellectual stimulation and individualized consideration, can increase employee motivation (Fernet et al., 2015), job satisfaction (Arnold, 2017) and ultimately organizational productivity (Kilroy et al., 2020). However, the current study takes a more nuanced approach by examining the role of transformational leadership within the specific context of human resource activities, with a particular focus on high-involvement work practices and their impact on elder employees’ innovation performance. The results of this study present a more complex picture, suggesting that the effects of transformational leadership are not universally positive. This is consistent with the arguments of some scholars who have suggested potential limitations and drawbacks of transformational leadership (Guay and Choi, 2015; Arnold, 2017; Jiang and Chen, 2021). In high-involvement work settings, transformational leadership may inadvertently dampen the autonomy and creativity that these practices aim to enhance, particularly among elder employees. This finding provides further evidence for the contextual application of high-involvement work practices in enhancing the innovation performance of elder employees.

### Practical implications

First and foremost, it is important to recognize that the high-involvement work practices adopted by organizations have a significant impact on the innovation behavior and performance of employees, especially older ones. This means devoting more time and resources to fostering a high-involvement work environment. Organizational leaders should set clear and achievable goals for elder employees encourage them to actively engage in intergenerational communication with other team members, and provide targeted training programs (Miao and Wu, 2021). These programs should be designed to equip elder employees with the necessary knowledge and skills that are aligned with their job roles and innovation opportunities (Bosch-Farré et al., 2020). In addition, managers should continuously seek ways to stimulate elder employees’ innovation motivation by recognizing their unique experiences and insights. In this way, organizations can fully utilize the potential of elder employees, thereby enriching the overall innovation capacity of the organization.

Second, the study shows the importance of innovative ways to employees’ innovative performance and different innovative ways have different effects on performance. The enterprise management personnel can combine own resources, the condition choice suitable innovation way. For enterprises with weak resources and capabilities, in order to improve short-term innovation efficiency, they can first choose utilization-type innovation, and gradually turn to explore the way of innovation with the improvement of resources and capabilities, improve competitiveness and increase revenue from innovation income.

Finally, companies can improve their management style to play a positive role in high-involvement work practices. For example, companies should pay attention to the choice of leadership style and develop their own innovation behavior to promote elder employees’ innovation performance according to different management and innovation situations. For example, improving transformational leadership for exploitative innovation and reducing transformational leadership for exploratory innovation. Master the coordination of innovation and leadership in innovation management practice.

### Limitations

Although this study follows scientific procedures in terms of model construction and research design, there are still some shortcomings due to resource constraints and other factors: First, due to geographical constraints, this study selected only some enterprises in Shandong Province for questionnaire survey, and the empirical results of enterprises in different regions may be different. Future studies can expand the distribution channels of questionnaires, increase the sample size and further analyze more random, diverse and universal samples to improve the universality of the study. Second, the employee self-assessment method was used to conduct the questionnaire survey. Although the three-stage questionnaire design can reduce the homogeneity error to some extent, the influence of individual subjective factors cannot be completely excluded. Future studies could try to use other assessments than the questionnaire survey and the up-down matching survey to reduce the homogeneity error through more objective data. Finally, methodological limitations lead to results that may not be convincing. Future studies can further explore the influence mechanism of high-involvement work practices on elder employees’ innovation performance by combining experimental studies and full sample surveys, and by comparing data at different stages.

## Data availability statement

The original contributions presented in the study are included in the article/supplementary material, further inquiries can be directed to the corresponding author.

## Author contributions

DJ: Conceptualization, Methodology, Writing – review & editing. YZ: Data curation, Investigation, Writing – original draft. HZ: Writing – review & editing. XW: Resources, Supervision, Writing – original draft.
